# Depletion of circulating IgM memory B cells predicts unfavourable outcome in COVID-19

**DOI:** 10.1038/s41598-020-77945-8

**Published:** 2020-11-30

**Authors:** Marco Vincenzo Lenti, Nicola Aronico, Ivan Pellegrino, Emanuela Boveri, Paolo Giuffrida, Federica Borrelli de Andreis, Patrizia Morbini, Laura Vanelli, Alessandra Pasini, Cristina Ubezio, Federica Melazzini, Alessandro Rascaroli, Valentina Antoci, Stefania Merli, Francesco Di Terlizzi, Umberto Sabatini, Ginevra Cambiè, Annamaria Tenore, Cristina Picone, Alessandro Vanoli, Luca Arcaini, Fausto Baldanti, Marco Paulli, Gino Roberto Corazza, Antonio Di Sabatino

**Affiliations:** 1 Department of Internal Medicine, San Matteo Hospital Foundation, University of Pavia, Pavia, Italy; 2 Anatomic Pathology Unit, Department of Molecular Medicine, San Matteo Hospital Foundation, University of Pavia, Pavia, Italy; 3 Division of Hematology, San Matteo Hospital Foundation, University of Pavia, Pavia, Italy; 4 Molecular Virology Unit, Microbiology and Virology Department, San Matteo Hospital Foundation, Pavia, Italy; 5 Clinica Medica, Fondazione IRCCS Policlinico San Matteo, Università Di Pavia, Piazzale Golgi 19, 27100 Pavia, Italy

**Keywords:** Adaptive immunity, Infectious diseases, Lymphocytes, Immunopathogenesis

## Abstract

Impaired immune responses have been hypothesised to be a possible trigger of unfavourable outcomes in coronavirus disease 2019 (COVID-19). We aimed to characterise IgM memory B cells in patients with COVID-19 admitted to an internal medicine ward in Northern Italy. Overall, 66 COVID-19 patients (mean age 74 ± 16.6 years; 29 females) were enrolled. Three patients (4.5%; 1 female) had been splenectomised and were excluded from further analyses. Fifty-five patients (87.3%) had IgM memory B cell depletion, and 18 (28.6%) died during hospitalisation (cumulative incidence rate 9.26/100 person-week; 5.8–14.7 95% CI). All patients who died had IgM memory B cell depletion. A superimposed infection was found in 6 patients (9.5%), all of them having IgM memory B cell depletion (cumulative incidence rate 3.08/100 person-week; 1.3–6.8 95% CI). At bivariable analyses, older age, sex, number of comorbidities, and peripheral blood lymphocyte count < 1500/µl were not correlated with IgM memory B cell depletion. A discrete-to-marked reduction of the B-cell compartment was also noticed in autoptic spleen specimens of two COVID-19 patients. We conclude that IgM memory B cells are commonly depleted in COVID-19 patients and this correlates with increased mortality and superimposed infections.

## Introduction

Coronavirus disease 2019 (COVID-19), caused by severe acute respiratory syndrome coronavirus 2 (SARS-CoV-2), is a zoonotic infectious disease that has become a global major threat in a matter of months^1–5^. Northern Italy has been particularly affected by the rapid spread of COVID-19 in March–May 2020^6^, and this has put a lot of pressure on the healthcare system, forcing to a rapid reorganisation of patients’ admission and care^7,8^. The immunological mechanisms leading to COVID-19 manifestations are still largely unknown.

SARSV-CoV-2 is an enveloped, positive-sense, single-stranded RNA virus, and similarly to SARS-CoV^9^, its pathogenicity seems to be related to the expression of the angiotensin-converting enzyme 2 receptor^10,11^, which mediates cell invasion, and to the immunologic host response characterised by an increased production of pro-inflammatory cytokines, such as interleukin-6 and tumour necrosis factor-α^12^. The decrease and exhaustion of circulating T cells have been hypothesised to lead disease progression, and counts of total T cells, either CD8+ or CD4+, were found to be negatively correlated with patient survival^13^.

On the contrary, little is known regarding humoral response to SARS-CoV-2, and B cells have been poorly characterised in this setting. In particular, human IgM + IgD + CD27 + B lymphocytes, also known as IgM memory B cells, represent a large subpopulation of the human B-cell pool that can rapidly differentiate into plasma cells, and play a major role in early inflammatory responses, including those caused by viral and bacterial infections^14,15^. The spleen, specifically its marginal zone, is crucial for preserving the IgM memory B-cell pool which plays a central role in mounting the immune response against encapsulated bacteria, thus preventing infections by *S. pneumoniae*, *N. meningitidis*, and *H. influenzae*^16^. Also, the role of IgM memory B cells in determining an effective immune response against viral infections, including influenza and HIV, is increasingly recognised in both humans and experimental models^17^. Indeed, patients without a spleen or with spleen hypofunction (i.e., hyposplenism) have lower number of circulating IgM memory B cells, and this is associated with worse health-related outcomes, including greater risk of developing serious infections or overwhelming post-splenectomy infections^18^.

The tropism of coronaviruses for the spleen has already been shown in patients infected by SARS-CoV and in an experimental model of Middle East respiratory syndrome^19,20^. Notably, spleen atrophy, mainly affecting the white pulp, has been recently observed in post-mortem cases of COVID-19^21–24^. More in depth, highest SARS-CoV-2 RNA levels were observed, among other organs, in the spleen, supporting the role of leukocytes as a route of dissemination of the virus from the airways to lymphoid organs^23^. Also, in a series of 11 autopsies of COVID-19 patients, atrophy of the white pulp due to lymphocyte depletion was noticed in ten cases, while the bone marrow never showed substantial changes^24^. Despite these studies point at a possible role of spleen function impairment, the influence of SARS-CoV-2 on spleen function and IgM memory B cells in a clinical setting is still unknown, neither is available information regarding the impact of COVID-19 in asplenic or hyposplenic patients.

Starting from these premises, we aimed to assess spleen function through circulating IgM memory B cells and pitted red cells (PRC)-red cells with membrane abnormalities (pits) visible under interference phase microscopy-in patients with COVID-19, in relation to their clinical outcome. Also, we explored histopathological alterations of autoptic spleen specimens collected from COVID-19 patients.

## Methods

### Study populations

This is a single-centre, longitudinal, prospective, study conducted in an academic, tertiary referral hospital from Northern Italy (San Matteo Hospital Foundation, Pavia), located close to the first COVID-19 outbreak which occurred earlier in February in Codogno (about 40 km from Pavia). We enrolled consecutive adult patients with a certain diagnosis of COVID-19 from March to May 2020, who were admitted to our internal medicine unit. During the SARS-CoV-2 Italian epidemic, the internal medicine unit was transformed into a “Covid” ward, in order to overcome the rapid increase of infected patients needing hospitalisation^7^. The diagnosis of COVID-19 was primarily based on both clinical (i.e., flu-like symptoms, fever, tachypnoea, hypoxemia, radiological interstitial pneumonia) and biological grounds (evidence of SARS-CoV-2 in nasopharyngeal swab or bronchoalveolar lavage). In particular, total nucleic acids (DNA/RNA) were extracted from samples (200 µl) using the QIAsymphony instrument with QIAsymphony DSP Virus/Pathogen Midi Kit (Complex 400 protocol) following the manufacturer’s instructions (QIAGEN, Qiagen, Hilden, Germany). Specific real-time PCR targeting RNA-dependent RNA polymerase and E genes were used to detect the presence of SARS-CoV-2 according to internationally recognised criteria^25,26^. Denial of informed consent, prognosis of less than 48 h, uncertain COVID-19 diagnosis, and inability to express consent (e.g., advanced dementia, coma) were the only exclusion criteria. All blood tests included in this study were requested as per clinical need and performed within 72 h from hospital admission.

As control groups for comparison of IgM memory B cells and PRC, we also enrolled 25 healthy volunteers (HV; mean age 50 ± 16 years; 9 females), 25 patients suffering from spleen hypofunction (mean age 45 ± 13 years; 14 females), and 20 patients who underwent splenectomy for trauma (mean age 45 ± 12 years; 8 females). HV had no history of chronic diseases. The spleen hypofunction group included ten patients with celiac disease (five treated and five not treated with a gluten-free diet), five with refractory celiac disease and ten with inflammatory bowel disease (five Crohn’s disease and five ulcerative colitis), all disorders known to be associated with functional hyposplenism^27–30^. Further, PRC data from 12 patients (mean age 58 ± 12 years; 5 females) splenectomised for reasons other than trauma, collected before splenectomy, and 20, 40, 60, 80, 120 days after splenectomy, were provided to show the PRC trend over time after spleen removal.

Finally, in order to evaluate the impact of COVID-19 on patients without a spleen or with splenic hypofunction, we administered a questionnaire by phone call to 140 asplenic/hyposplenic patients regarding possible COVID-19 symptoms and diagnosis, if any, that occurred in the previous 3 months, corresponding to the beginning of the outbreak in Lombardy. Of 140 patients who were contacted, 67 (mean age 61.1 ± 16.2; 29 females) completed the questionnaire. The questionnaire included demographic variables, contact history, COVID-19 symptoms, confirmed COVID-19 diagnosis and hospitalisation. For comparison, a sample of 400 (mean age 63.2 ± 15.2, 219 females) non-asplenic/non-hyposplenic unselected individuals from the same geographical area was administered the same questionnaire.

The study was approved by the local ethics committee (San Matteo Hospital Foundation, Pavia) on March 13th, 2020, and informed consent for study participation and publication of anonymised data was obtained from all participants. The results of this study are reported according to the STrengthening the Reporting of OBservational studies in Epidemiology (STROBE) recommendations for quality assurance. All methods were performed in accordance with the relevant guidelines and regulations.

### Evaluation of peripheral B cell subsets

Peripheral blood lymphocytes were isolated from heparinized blood by Lymphoprep gradient centrifugation (Nicamed, Oslo, Norway). For detecting IgM memory B cells and switched memory B cells, peripheral blood cells were stained with the appropriate antibody combination, including IgM FITC, CD24 PE, CD38 PERCP Cy5.5, CD19PECy7, CD27 APC, IgG APC H7, IgD V450, CD45 V500C (Becton Dickinson Co., San Jose, CA). All flow cytometric analyses were performed on a FACSLyric system interfaced to a BD FACSuite (Becton Dickinson Co.) computer program. Dead cells were excluded from analysis by side/forward scatter gating. The lower limit of normal for IgM memory B cells was 26/µl, as previously reported^31^. Gating strategies for the detection of lymphocyte subpopulations are depicted in Supplementary Fig. 1.

### Evaluation of circulating pitted red cells

Splenic function was assessed by counting PRC (i.e., erythrocytes with membrane abnormalities visible under interference phase microscopy, also referred to as *pits*), which is the most accurate and reproducible test for this purpose^16^. In brief, a drop of fresh venous blood was mixed with 1.5 ml 3% buffered-glutaraldehyde solution with a pH of 7.4. One drop of this solution was placed on a clean slide, preparing a blood smear. One thousand red cells were examined in a wet preparation (magnification, 1000X) with a direct-interference contrast microscope (Leitz Dialux 20; Lietz, Cape Coral, FL) equipped with Nomarsky optics by two independent observers who were unaware of patients’ diagnoses. The mean percentage of PRC between the two observers was calculated and taken as a measure of splenic function (upper limit of normal 4%). In case of a discrepancy > 1% between the observers, the blood smear was assessed again, until concordance was reached. A representative picture of PRC is shown in Supplementary Fig. 2.

### Autoptic spleen specimens

Autoptic spleen specimens were available for two patients (one male, 69 years old, and one female, 71 years old) who died from COVID-19. In these two cases, spleen specimens were collected during minimally invasive autopsies. Specimens were formalin-fixed and paraffin-embedded. Three-millimeter-thick paraffin sections were used for morphology (hematoxylin–eosin staining) and immunohistochemistry by Dako Omnis automatic platform (Agilent, Santa Clara, CA). Spleen specimens obtained from a non- COVID-19, 58-year old man, who had undergone abdominal surgery for extra-splenic causes, were used as control. Immunohistochemistry with monoclonal antibodies anti-CD79a, anti-CD3, and anti-CD68R was performed for staining B- and T-cell spleen areas and histiocytes, respectively.

### Statistical analysis

The sample size was not calculated a priori*,* given the explorative nature of the study. For clinical features, a descriptive statistical analysis was performed, and, when appropriate, data were expressed as number of total and/or percentage, mean and standard deviation (SD), or median and range or interquartile range (IQR). For percentage calculation, patients were excluded when variables were missing. At univariable analysis, Wilcoxon matched-pairs sign rank test was used for comparison amongst groups. For relevant laboratory parameters, Spearman’s correlation coefficient was calculated, together with its 95% confidence interval (95% CI). For all correlations, COVID-19 patients who had been splenectomised were excluded from the analyses. Univariable and bivariable Cox regression models were fitted. Hazards ratios (HR) and 95% CI were computed. Kaplan Meier event-free survival (death) was computed and plotted for patients who had low IgM B memory cells versus those who had normal ranges. For the analyses of mortality, the variable “low IgM memory B cell” was adjusted for the following parameters in bivariable models: age greater than 58 years, male sex, PRC greater than 4%, three or more than three associated chronic diseases, and a number of total lymphocytes lower than 1500/µl. Finally, HR for infectious events in patients with low IgM memory B cells was evaluated. Two-tailed p values less than 0.05 were considered statistically significant. The software STATA 16 (StataCorp, College Station, TX) was used for all computations.

## Results

Over the study period, a definite diagnosis of COVID-19 was made in 197 patients. Of these, 66 (mean age 74 ± 16.6 years; 29 females) took part to the study (Table 1), while the others were excluded because had a prognosis of less than 48 h, denied consent, or had incomplete data. The median time lapse from COVID-19 symptom onset to hospital admission was 10 days (IQR 6–14 days).

**Table 1:** Demographic and clinical characteristics of the overall cohort of 66 COVID-19 patients.

	Patients (n = 66)
Age (years), mean (SD)	74 (16.6)
**Sex, n (%)**	
Female	29 (43.9)
Comorbidities, mean (SD)	3 (2)
**At least one chronic associated disease, n (%)**	59 (89.4)
Essential hypertension	32 (48.5)
Chronic heart failure	26 (39.4)
Obesity	16 (24.2)
Type 2 diabetes mellitus	14 (21.2)
Chronic kidney failure	14 (21.2)
Chronic liver disease	9 (13.6)
Autoimmune diseases	8 (12.1)
Chronic obstructive pulmonary disease	6 (9.1)
Active neoplasia	2 (3.0)
Previous splenectomy	3 (4.5)

*SD* standard deviation.

Notably, 49/66 (74.2%) patients were aged more than 65 years old and suffered from multimorbidity. Overall, 3/66 (4.5%) patients had been splenectomised for trauma and their characteristics and outcome are reported in Table 2. These three splenectomised patients were not included in the following analyses, so that from here onwards all analyses focused on the remaining 63 patients whose laboratory tests are reported in Supplementary Table 1. Notably, 55/63 patients (87.3%) had IgM memory B cell depletion (≤ 26/µl), while 10/63 patients (15.8%) had PRC counts indicative of defective spleen function (> 4%). Also, considering the normal reference value of circulating plasmablasts (< 2–3 n/µl), all patients showed the expansion of this B-cell subset^32^. Among patients who had increased PRC, none had a possible predisposing cause of functional hyposplenism. Spearman’s correlation coefficients of different laboratory tests are shown in Supplementary Table 2. Circulating IgM memory B cells turned out to significantly and positively correlate with haemoglobin levels and neutrophil numbers, whereas no correlation was found with the other parameters, including systemic inflammatory indexes.

**Table 2:** Main clinical and laboratory characteristics of the three COVID-19 splenectomised patients.

Patient	#1	#2	#3
Age	83	53	55
Sex	F	M	M
Age at splenectomy (years)	58	41	29
Reason for splenectomy	Trauma	Trauma	Trauma
Length of stay (days)	12	18	9
Infection during stay	No	UTI (*E. coli*)	No
Total lymphocytes (n/µl)	400	1588	1522
Total B cells (n/µl)	69.2	492.4	156.8
Memory B cells (n/µl)	3.2	43.8	24.8
IgM memory B cells (n/µl)	1.1	12.8	5.02
Switched memory B cells (n/µl)	2.5	27.1	14.6
Platelet-to-lymphocyte ratio	0.9	0.1	0.3
Neutrophil-to-lymphocyte ratio	22.9	6.4	2.6
Lactate dehydrogenase (mU/ml)	408	222	265
C reactive protein (mg/dl)	15.3	10.3	5.3
Pitted red cells (%)	6.4	10.2	12.4
Platelets (× 10^3^/µl)	374	266	544
Outcome	Discharged	Discharged	Dead
Venous thromboembolism	No	No	No

*UTI* urine tract infection.

**Figure 1:**
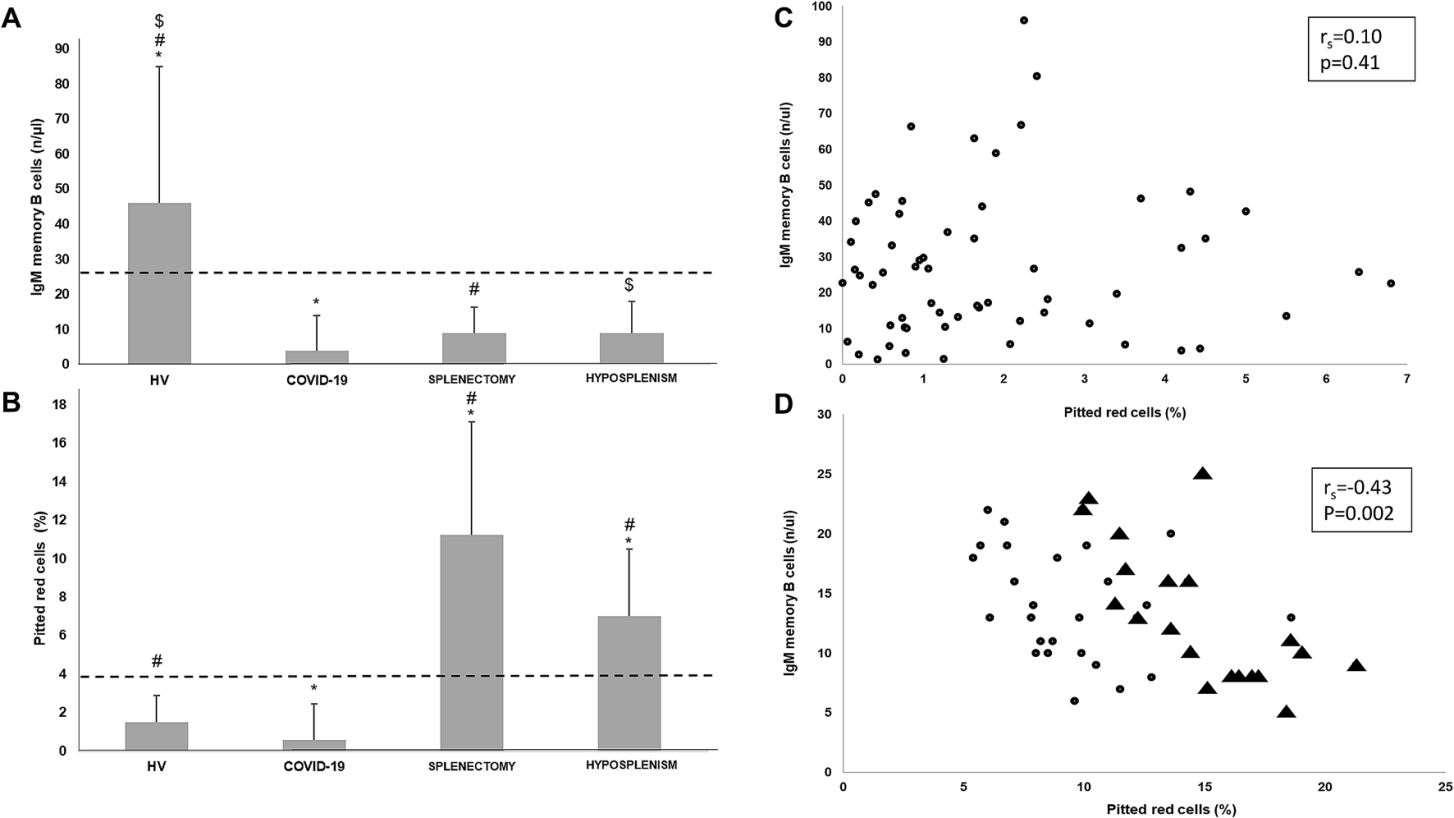
(**A**, **B**) Bar charts showing median, 75th percentile, and statistical significance of IgM memory B cells (**A**) and pitted red cell (PRC; **B**) count of patients with coronavirus disease 2019 (COVID-19) *vs* healthy volunteers (HV), splenectomised patients, and patients with spleen hypofunction. The dashed lines indicate the lower normal value of IgM memory B cells (26/µl) and the upper limit of normal of PRC count (4%), respectively. (**A**) *^#$^p < 0.01; (**B**) *^#^p < 0.01. (**C**, **D**) Spearman’s correlation coefficient between pitted red cell (PRC) count (%) and IgM memory B cells (n/µ) in COVID-19 patients (**C**) compared to hyposplenic (circles) and asplenic (triangles) patients (**D**). In the COVID-19 group, three patients with IgM memory B cell count greater than 100/µl were removed (outliers).

Figure 1 shows the median circulating IgM memory B cell count (A) and PRC (B) in cases compared to control groups. Notably, COVID-19 patients had significantly (p < 0.01) lower circulating IgM memory B cell numbers (median 5.9/µl, IQR 2.1–13.9/µl) compared to HV (median 65.0/µl, IQR 51.0–85.0/µl). As expected, asplenic and hyposplenic patients showed significantly (p < 0.01) lower numbers of circulating IgM memory B cells (median 12.0/µl, IQR 8.0–16.5/µl and median 13.0/µl, IQR 10.0–18.0/µl, respectively) in comparison to HV. No significant difference was observed between COVID-19 patients and both asplenic and hyposplenic patients. Regarding switched memory B cells, no significant difference was observed between the four groups studied (data not shown). No significant difference was observed in PRC count between COVID-19 patients (median 1.3%, IQR 0.7–2.4%) and HV (median 2.1%, IQR 1.1–2.9%). Asplenic and hyposplenic patients had significantly (p < 0.01) higher PRC counts (median 14.9%, IQR 13.5–17.1% and median 8.7%, IQR 7.1–10.5%, respectively) compared to both COVID-19 patients and HV.

Correlation between circulating IgM memory B cells and PRC (scatter plots, Spearman’s coefficient) in COVID-19 patients versus asplenic and hyposplenic patients is shown in Fig. 1C,D, respectively. Spearman’s correlation coefficient did not show a statistical significance in patients with COVID-19 (Fig. 1C), while a substantial correlation was seen in asplenic and hyposplenic patients (Fig. 1D). The fact that the time needed for filtering function impairment (PRC > 4%) in a cohort of 12 electively splenectomised patients is almost 2 months (Supplementary Fig. 3) provides a physio-pathological explanation for the lack of PRC increase in COVID-19 patients, whose median disease duration was 10 days at hospital admission.

As regards the impact of SARS-CoV-2 infection on patients without a spleen or with defective spleen function, we found that only one male patient among the 67 interviewed patients (1.5%) had a proved COVID-19—i.e., supported by a positive nasopharyngeal swab—in comparison to 2/400 (0.5%; p = 0.34) of a control cohort. None of the two COVID-19 patients belonging to the control cohort were hospitalised, while the asplenic patient required admission to hospital and was later discharged.

**Figure 2:**
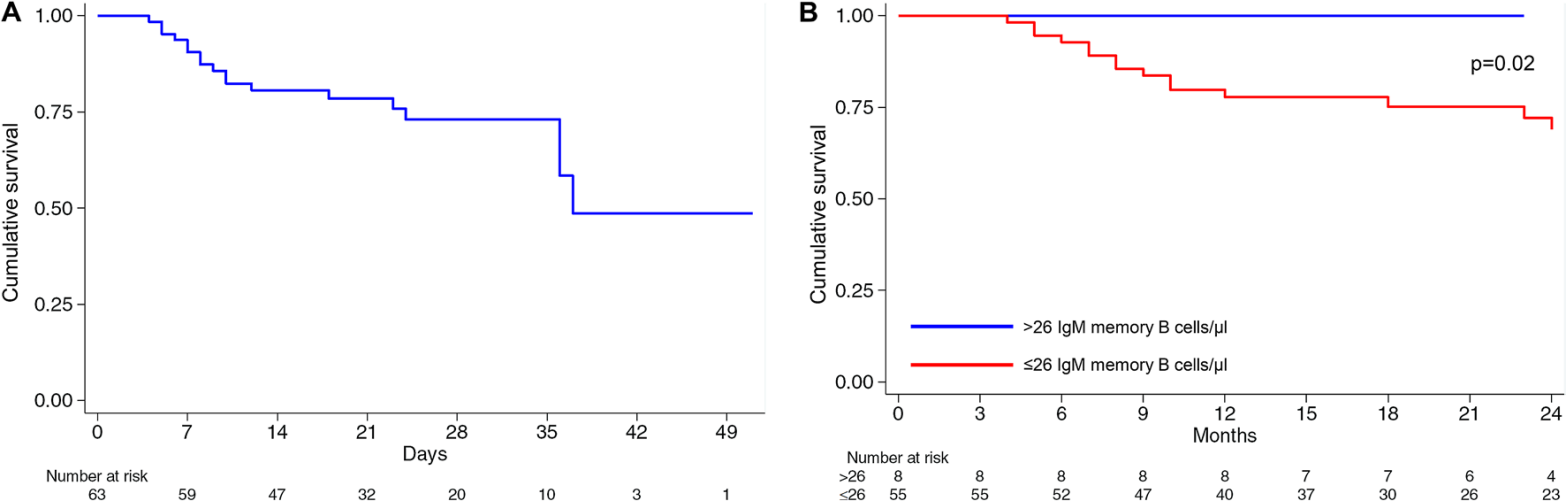
Kaplan Meier survival estimates. On the left-hand side (**A**), the whole cohort of patients with coronavirus disease 2019 are taken into account, while on the right-hand side (**B**) patients were divided according to the presence or absence of IgM memory B cell depletion.

Overall, 18/63 patients (28.6%) died during hospitalisation within a median time of 15 days (IQR 8–22), with a cumulative incidence rate of 9.26 per 100 person-week (5.8–14.7 95% CI). All patients who died had circulating IgM memory B cells ≤ 26/µl, hence the HR tends to infinity. When considering all patients with circulating IgM memory B cells ≤ 26/µl, the cumulative incidence rate of death was 11.08 per 100 person-week (6.9–19.5 95% CI). The Kaplan Meier survival curves (overall, panel A, and according to IgM memory B cell cut-off, panel B) are shown in Fig. 2. Of note, a significantly (p = 0.02) lower survival probability was observed in COVID-19 patients with circulating IgM memory B cells ≤ 26/µl in comparison to patients with normal circulating IgM memory B cell numbers. Table 3 shows different bivariable models for the outcome “death”. The presence of more than three associated chronic disorders is just below statistical significance, while all other adjusting factors are not statistically associated with the outcome.

**Table 3:** Bivariable analyses for adjusting mortality in the 55 COVID-19 patients with ≤ 26/µl circulating IgM memory B cells.

Variables	HR	95% CI	p-value
Age > 58 years	4.04	0.31–0.85	0.182
Sex (male)	0.91	0.36–2.30	0.838
Pitted red cells > 4%	1.39	0.44–4.34	0.575
Associated chronic disorders ≥ 3	2.59	0.92–7.28	0.071
Lymphocytes < 1500/µl	1.53	0.18–13.26	0.699

*CI* confidence interval, *HR* hazard ratio.

On the basis of the crucial role of IgM memory B cells in controlling bacterial infections, we evaluated the occurrence of infectious events in our COVID-19 cohort. A superimposed infection, mostly affecting the urinary tract, was found in 6/63 patients (9.5%), all of them having circulating IgM memory B cells ≤ 26/µl (Table 4). Four of these six patients developed a sepsis. As regards the outcome, two of them died, one required admission to the intensive care unit, and three recovered. The cumulative incidence rate of infections in the overall cohort of 63 COVID-19 patients was 3.08 per 100 person-week (1.3–6.8 95% CI), while in those having circulating IgM memory B cells ≤ 26/µl, it was 3.69 per 100 person-week (1.6–8.2 95% CI).

**Table 4:** Main clinical characteristics of the six COVID-19 patients who developed superimposed infections.

Patient	#1	#2	#3	#4	#5	#6
Age (years)	89	88	88	82	28	86
Sex	Male	Female	Female	Male	Female	Male
Length of stay (days)	16	18	28	25	42	4
Site of isolation	Urine	Urine	Urine	Skin	Urine, blood	Urine
Microorganism(s)	*E. coli*	*E. faecalis*	*E. coli*, *C. glabrata*	*S. aureus*	*E. faecalis*	*E. faecalis*
Sepsis	No	Yes	No	Yes	Yes	Yes
Total lymphocytes (n/µl)	963.0	829.5	582.5	1148.4	767.4	1022.5
Total B cells (n/µl)	27.9	145.1	41.9	28.7	55.2	71.5
Memory B cells (n/µl)	19.6	46.3	26.6	6.3	3.9	42.7
IgM memory B cells (n/µl)	6.4	7.9	9.7	1.8	1.6	1.5
Switched memory B cells (n/µl)	10.7	30.1	13.9	4.5	1.8	15.0
Platelet-to-lymphocyte ratio	0.23	0.51	0.34	0.22	0.23	0.21
Neutrophil-to-lymphocyte ratio	7.69	14.41	18.81	2.92	2.84	6.27
Lactate dehydrogenase (mU/ml)	170	217	330	251	250	637
C reactive protein (mg/dl)	15.5	13.5	7.2	3.2	3.7	19.1
Pitted red cells (%)	3.4	3.7	2.3	0.06	4.2	5.0
Platelets (× 10^3^/µl)	223	425	197	249	179	218
Outcome	Discharged	Dead	Discharged	ICU	Discharged	Dead

*ICU* intensive care unit.

In a sub-cohort of COVID-19 patients who had IgM memory B cell depletion, we evaluated both circulating IgM memory B cells (eight patients) and PRC (13 patients) after a median time of 25 days (IQR 21–32) since hospital admission. No statistically significant difference was seen between baseline and follow-up for both variables (data not shown).

**Figure 3:**
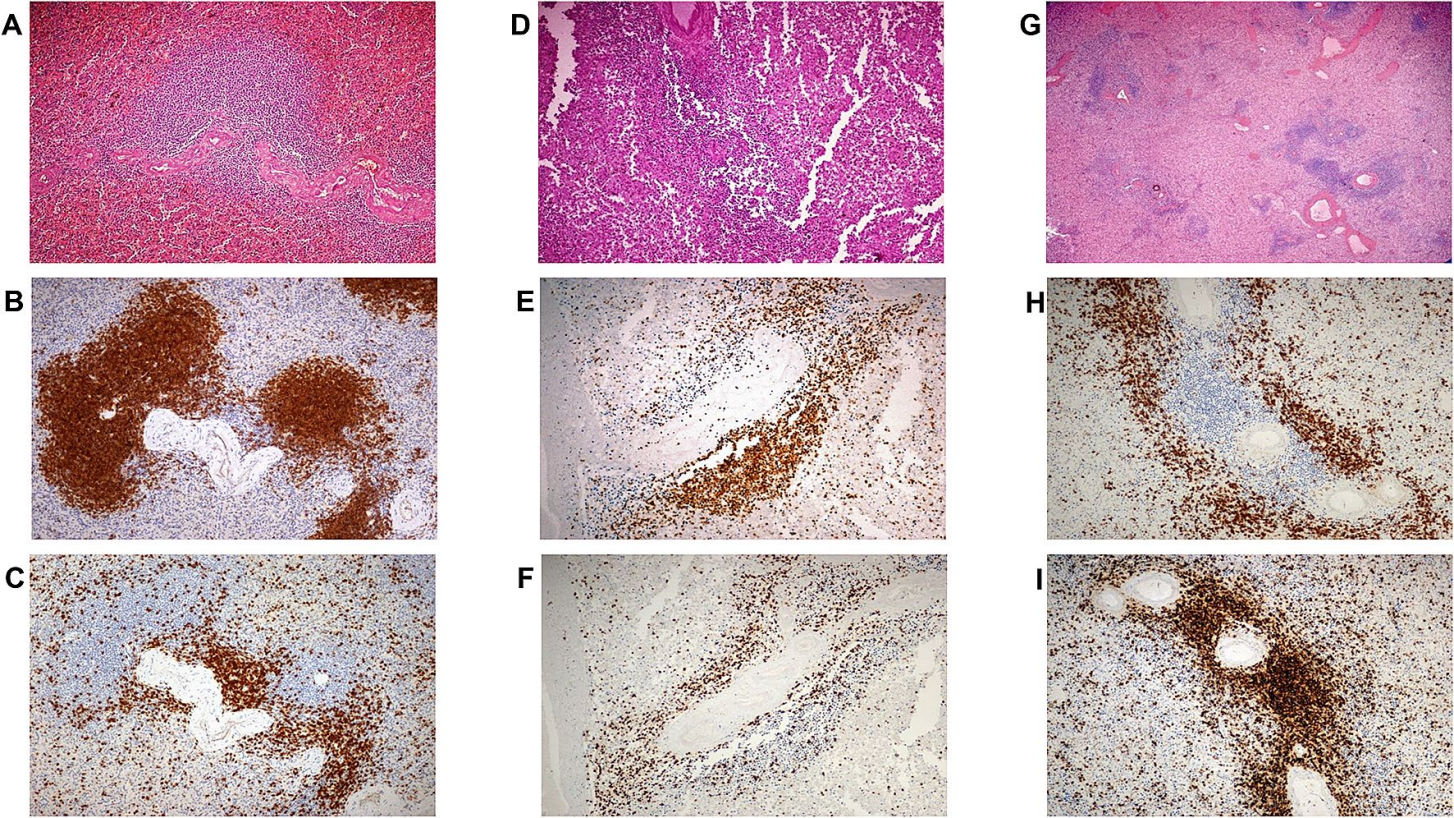
Normal spleen architecture (male, 58 years old, splenectomised during pancreatic surgery). (**A**) usual representation of the white and red pulp (HE, ×20); immunohistochemistry with antibodies anti-CD79a (**B**) and anti-CD3 (**C**) demonstrates normal distribution of B and T cell areas around vessels (SABC, 20x, Dako Omnis automatic platform). Spleen parenchyma in minimally invasive autopsies of COVID-19 infected patients: **D**–**F**) male, 69 years old; **G**–**I**) female, 71 years old; **D**, **G**) white pulp appears diminished (HE, ×10) due to reduction of both B CD79a+ (**E**, **H**) and T CD3+ areas (**F**, **I**) (**E**, **F**, **H**, **I**: SABC 20x, Dako Omnis automatic platform).

Finally, since spleen white pulp is essential for the integrity of the IgM memory B cell pool, we investigated autoptic spleen specimens obtained from two patients who died from COVID-19 (Fig. 3). In comparison to the spleen of the control individual, the splenic parenchyma of the two COVID-19 patients showed a discrete-to-marked reduction of the white pulp associated with a more severe depletion of the B-cell compartment compared to the T-cell one. Moreover, the red pulp turned out to be expanded and congested, with focal, non-confluent micro-haemorrhagic areas and scattered CD68R+ histiocytes without features of erythro- and/or hemo-phagocytosis. No vascular thrombi, nor significant fibrosis, were noticed.

## Discussion

We have herein found a high prevalence of IgM memory B cell depletion in a large and well-characterised cohort of patients with COVID-19. IgM memory B cell depletion was associated with worse outcomes, including a higher mortality rate and a higher risk of developing superimposed infections. Contrary to what happens in asplenia and spleen hypofunction, in which a negative correlation between circulating IgM memory B cells and PRC is noticed, COVID-19 is characterised by a dissociation between the immune and filtering functions of the spleen. In keeping with the results obtained on circulating IgM memory B cells, the B-cell areas of the spleen are obliterated. Finally, in our high-risk area, asplenic/hyposplenic patients do not appear to have an increased risk of developing COVID-19.

All patients who died during hospitalisation or who developed superimposed infections showed depletion of IgM memory B cells. COVID-19 patients seem to be functionally splenectomised. In fact, in our cohort of 63 COVID-19 patients, IgM memory B cell depletion appears to be as severe as that of splenectomised patients, although differing from the latter due to the lack of association between immunological and hemocateretic functions. In particular, patients with low number of IgM memory B cells had an increased mortality rate (not affected by age, gender, total number of lymphocytes, PRC). Notably, at bivariable analysis, total lymphocyte depletion was not independently associated with mortality. Hence, IgM memory B cell depletion per se, and not lymphocytopenia, was responsible for the observed outcome. Considering that IgM memory B cells are able to initiate the immune response against other viral infections^17^, they could have a role in limiting the spread of SARS-CoV-2 to other organs, or they could limit the abnormal “cytokine storm” which is responsible for additional organ damage^33^. A deep immune perturbation of both the innate and adaptive immunity has already been described in patients with severe COVID-19, characterised by T-cell activation, lymphocytopenia, and altered neutrophile to lymphocyte ratio^34^. Notably, we herein confirm the marked expansion of the plasmablasts, which we noticed in our patients who had moderate-severe COVID-19. This feature is also noticed in other viral infections, such as those caused by HIV and Ebola virus^35^. Plasmablasts are immature B cells which are essential for initiating a long-lasting immune response and memory against infections, even if their significance in COVID-19 still needs to be ascertained.

We also found an increased infectious risk in our 63-patient cohort, in the majority of cases sustained by bacteria spreading from the gastrointestinal tract. This is in keeping with the evidence that subjects who have IgM memory B cell depletion are characterised by lack of mucosal IgA-secreting plasma-cells, an important factor of innate immunity that prevents gut-borne infections and contributes to the normal immunosurveillance function of the gut^36^. Although overwhelming post-splenectomy infections are more frequently caused by encapsulated bacteria, such as *S. pneumoniae, H. influenzae,* and *N. meningitidis*, other pathogens may be cause of this condition. Our patients showed infections mainly sustained by *E. coli* and *E. faecalis* and these pathogens are frequently implicated in the development of sepsis in splenectomised patients^37^. Indeed, most of our patients were elderly patients with multimorbidity, some of them requiring a urinary catheter and/or central venous catheter, that could favour the spread of bacteria. However, this reflects the setting of enrolment, which is that of an internal medicine unit where complex, multimorbid patients are usually admitted^38^.

Impairment of immunological spleen function appears to be persistent, at least after a median follow-up time of 25 days, and hemocateretic function measured with PRC count is still preserved. We have shown that PRC need roughly 60 days before appearing in the bloodstream after splenectomy, as a consequence of the median half-life of the erythrocytes, hence a follow-up of 25 days could be too short for proving changes in the PRC count, if any. Unfortunately, due to the ongoing epidemic, it was not possible to follow-up the patients for a longer time. Hence, whether spleen immunological defect is reversible or not after COVID-19 still needs to be ascertained.

In our cohort, we had the chance to assess three COVID-19 patients who had been previously splenectomised. As expected, all of them had IgM memory B cell depletion, and one of them died. Data regarding the impact of COVID-19 on patients with asplenia or spleen dysfunction are completely lacking, although the British Society of Haematology has drafted a document of recommendations for asplenic and hyposplenic patients, stating that there seems to be no reason that spleen function impairment might render these patients at higher risk of COVID-19^39^. Our phone call interview seems to corroborate this hypothesis, even if the small sample size does not allow to draw any firm conclusion. If we agree that asplenic patients do not seem to be more susceptible to COVID-19, whether they could be more exposed to its complications or to a higher mortality rate remains to be addressed.

Tropism for the spleen is not limited to RNA viruses implicated in the development of acute respiratory distress syndrome, such as SARS-CoV, SARS-CoV-2 and Middle East respiratory syndrome virus. For example, HIV is known to alter the spleen structure^40^ and function^41^ even if the pathogenic mechanisms underlying white pulp atrophy in HIV infected patients are not completely known^16^. As regards SARS-CoV-2, its tropism for the spleen could be justified by the expression of the ACE2 receptor in the red pulp sinus endothelium^42^.

Our study has indeed some limitations, including a rather small sample size compared to the magnitude of COVID-19 Italian epidemic, a relatively high mean patients’ age, a short-term follow-up, and the lack of in vitro experiments addressing IgM memory B cell function. However, it focuses for the first time on the function of an organ which is crucial in mounting the immune response against infections.

In conclusion, SARS-CoV-2 seems to affect spleen function by reducing the IgM memory B cell pool, and this is associated with adverse outcomes. Whether the tropism of SARS-CoV-2 for the spleen is involved in affecting IgM memory B cell numbers is yet to be defined. Further studies are needed to clarify the burden of SARS-CoV-2 in patients without a spleen or with spleen dysfunction. As an abnormal spleen function is implicated in the development of thromboembolic manifestations, it would also be interesting to verify whether this mechanism might contribute to the increased risk of thromboembolism observed in COVID-19 patients^43^.

## Supplementary information

Supplementary Information 1.

Supplementary Figure 1.

Supplementary Figure 2.

Supplementary Figure 3.

## Abbreviations

COVID-19: Coronavirus disease 2019
PRC: Pitted red cell
SARS-CoV-2: Severe acute respiratory syndrome coronavirus 2
