# Supplementary material for: Depletion of circulating IgM memory B cells predicts unfavourable outcome in COVID-19
Source: Sci Rep. 2020 Nov 30;10:20836. doi: 10.1038/s41598-020-77945-8 (PMC7705651; doi:10.1038/s41598-020-77945-8)
Supplement: Supplementary file 1 — Supplementary Information 1. [file 41598_2020_77945_MOESM1_ESM.pdf]

Depletion of circulating IgM memory B cells predicts unfavourable outcome in Covid-19

Running head: IgM memory B cell depletion in Covid-19

Marco Vincenzo Lenti[1], MD, Nicola Aronico[1], MD, Ivan Pellegrino[1], MD, Emanuela Boveri[2], MD, Paolo Giuffrida[1], MD, Federica Borrelli de Andreis[1], MD, Patrizia Morbini[2], MD, Laura Vanelli[3], MD, Alessandra Pasini[1], MD, Cristina Ubezio[1], MD, Federica Melazzini[1], PhD, Alessandro Rascaroli[1], MD, Valentina Antoci[1], MD, Stefania Merli[1], MD, Francesco Di Terlizzi[1], MD, Umberto Sabatini[1], MD, Ginevra Cambiè[1], MD, Annamaria Tenore[3], MD, Cristina Picone[3], MD, Alessandro Vanoli[2], PhD, Luca Arcaini[3], MD, Fausto Baldanti[4], MD, Marco Paulli[2], MD, Gino Roberto Corazza[1], MD, Antonio Di Sabatino,[1] MD

Supplementary Table 1. Laboratory results at the time of hospital admission of the 63 Covid-19 patients who entered into the final analyses

Variables	Patients (n=63)
**Haemoglobin** (g/dl), median (range)	11.8 (9.9-13.1)
Below normality range, n (%)	38/63 (60.3)
**MCV** (fl), median (range)	89.8 (86-91.8)
Below normality range, n (%)	4/63 (6.3)
Above normality range, n (%)	2/63 (3.15)
**RDW**, median (range)	15.1 (14.2-16.8)
Above normality range, n (%)	30/63 (47.6)
**Platelets** ($x10^3/\mu l$), median (range)	204 (140-251)
Below normality range, n (%)	17/63 (26.9)
Above normality range, n (%)	1/63 (1.6)
**MPV** (fl), median (range)	9.1 (8.4-19)
Below normality range, n (%)	7/63 (11.1)
Above normality range, n (%)	1/63 (1.6)
**Leukocytes** (n/µl), median (range)	7300 (5320-9600)
Below normality range, n (%)	9/63 (14.3)
Above normality range, n (%)	11/63 (17.4)
**Neutrophil-to-lymphocyte ratio**, median (range)	7.96 (4.99-12.43)
Above normality range, n (%)	56/63 (88.8)
**Neutrophils** (n/µl), median (range)	6110 (3900-8300)
Below normality range, n (%)	1/63 (1.6)
Above normality range, n (%)	26/63 (41.2)
**Total lymphocytes** (n/µl), median (range)	730 (550-940)
Below normality range, n (%)	58/63 (92.0)
**Eosinophils** (n/µl), median (range)	0 (0-20)
Below normality range, n (%)	56/63 (89.0)
**NK cells** (n/µl), median (range)	14.1 (10.5-18.4)
Below normality range, n (%)	2/53 (3.7)
**CD4+ T cells** (n/µl), median (range)	401 (278-582)
Below normality range, n (%)	31/53 (58.5)
**CD8+ T cells** (n/µl), median (range)	169 (114-304)
Above normality range, n (%)	52/53 (98.1)
**CD4/CD8 ratio**, median (range)	2.2 (1.1-3.7)
Below normality range, n (%)	4/53 (7.5)
Above normality range, n (%)	27/53 (50.9)
**Total B cells** (n/µl), median (range)	74.76 (42.86-130.94)
Below normality range, n (%)	13/63 (20.6)
**Memory B cells** (n/µl), median (range)	25.65 (12.09-42.73)
Below normality range, n (%)	55/63 (87.3)
**IgM memory B cells** (n/µl), median (range)	5.92 (1.9-14.48)
Below normality range, n (%)	55/63 (87.3)
**Switched memory B cells** (n/µl), median (range)	10.72 (5.64-18.16)
Below normality range, n (%)	49/63 (77.7)
**Plasmablasts** (n/µl), median (range)	10.9 (3.2-31)
**Pitted red cells** (%), median (range)	1.30 (0.72-2.45)
Above normality range, n (%)	10/63 (15.8)

**INR**, median (range)	1.10 (1.02-1.22)
Above normality range, n (%)	17/63 (27.0)
**C reactive protein** (mg/dl), median (range)	9.86 (4.69-15.57)
Above normality range, n (%)	60/63 (95.2)
**Procalcitonin** (ng/ml), median (range)	0.32 (0.07-1.08)
Above normality range, n (%)	44/56 (78.5)
**LDH** (mU/ml), median (range)	330 (255-426)
Above normality range, n (%)	50/63 (79.3)
**AST** (mU/ml), median (range)	35.5 (25-46)
Above normality range, n (%)	22/63 (34.9)
**ALT** (mU/ml), median (range)	20 (15-36)
Above normality range, n (%)	13/63 (20.6)
**Cholinesterase** (mU/ml), median (range)	5707 (4426-7167)
Below normality range, n (%)	17/60 (28.3)
**GGT** (mU/ml), median (range)	34 (21-56)
Above normality range, n (%)	27/63 (42.8)
**Total bilirubin** (mg/dl), median (range)	0.54 (0.41-0.99)
Below normality range, n (%)	7/63 (11.1)
Above normality range, n (%)	9/63 (14.2)
**Albumin** (g/dl), median (range)	2.8 (2.4-3.1)
Below normality range, n (%)	51/57 (89.5)
**D-dimer** (ng/ml), median (range)	1740 (810-4932)
Above normality range, n (%)	36/39 (92.3)
**PaO_2/FiO_2**, median (range)	266.5 (157.5-328.5)
Below normality range, n (%)	27/44 (61.3)

Abbreviations: ALT, alanine aminotransferase; AST, aspartate aminotransferase; GGT, gamma-glutamyl transpeptidase; INR, international normalized ratio; LDH, lactate dehydrogenase; MCV, mean corpuscular volume; MPV, mean platelet volume; NK, natural killer; PaO_2/FiO_2, arterial oxygen partial pressure to fractional inspired oxygen ratio; RDW, red cells distribution width.

Supplementary Table 2. Spearman's correlation coefficient between laboratory parameters in the 63 Covid-19 patients who entered in the final analyses

Variables	r_S	p-value
**IgM memory B cells (n/µl) vs PLT ($x10^3$/µl)**	0.219	0.083
**IgM memory B cells (n/µl) vs plasmablasts (n/µl)**	0.244	0.053
**IgM memory B cells (n/µl) vs NK cells (n/µl)**	-0.0059	0.962
**IgM memory B cells (n/µl) vs neutrophils (n/µl)**	0.386	0.0017
**IgM memory B cells (n/µl) vs CRP (mg/dl)**	0.218	0.084
**IgM memory B cells (n/µl) vs LDH (mU/ml)**	0.0745	0.561
**IgM memory B cells (n/µl) vs D-dimer (ng/ml)**	-0.080	0.625
**IgM memory B cells (n/µl) vs total lymphocytes (n/µl)**	0.507	2.193
**IgM memory B cells (n/µl) vs T cells (n/µl)**	0.553	1.752
**IgM memory B cells (n/µl) vs haemoglobin (g/dl)**	0.252	0.045
**IgM memory B cells (n/µl) vs albumin (g/dl)**	-0.112	0.407

Abbreviations: CRP, C reactive protein; LDH, lactate dehydrogenase; NK, natural killer; PLT, platelet.

Supplementary Figure 1. Identification of mature, IgM memory and switched memory B cells. A) doublet exclusion; B) CD45+ cells are included; C) lymphomonocytes are included; D) CD19+ cells are included; E) identification of transitional B cell(CD38++/CD24++); F) identification of plasmablasts (CD38++/CD27+); G) analysis exclusion of transitional and plasmablasts; expression of CD27 discriminates CD19+/CD27- mature B cells and CD19+/CD27+ memory B cells; H) memory B cells can be divided into IgM memory (IgM^{bright}/IgD^{dull}) and switched memory (IgM-/IgD-) B cells.

Supplementary Figure 2. Representative picture of two pitted red cells (direct-interference contrast microscope equipped with Nomarsky optics). Pits can be seen on the surface of the erythrocytes (black arrows).

Supplementary Figure 3. Trend over time of pitted red cells (PRC) in a cohort of patients who were splenectomised (day 0) for reasons other than trauma. PRC percentage increases over time and all patients showed increased PRC at day 40. The dashed line indicates the upper limit of normal PRC (4%). Statistical significance always refers to day 0.
