# Supplementary figures and images for: Depletion of circulating IgM memory B cells predicts unfavourable outcome in COVID-19
Source: Sci Rep. 2020 Nov 30;10:20836. doi: 10.1038/s41598-020-77945-8 (PMC7705651; doi:10.1038/s41598-020-77945-8)

## Slide 1
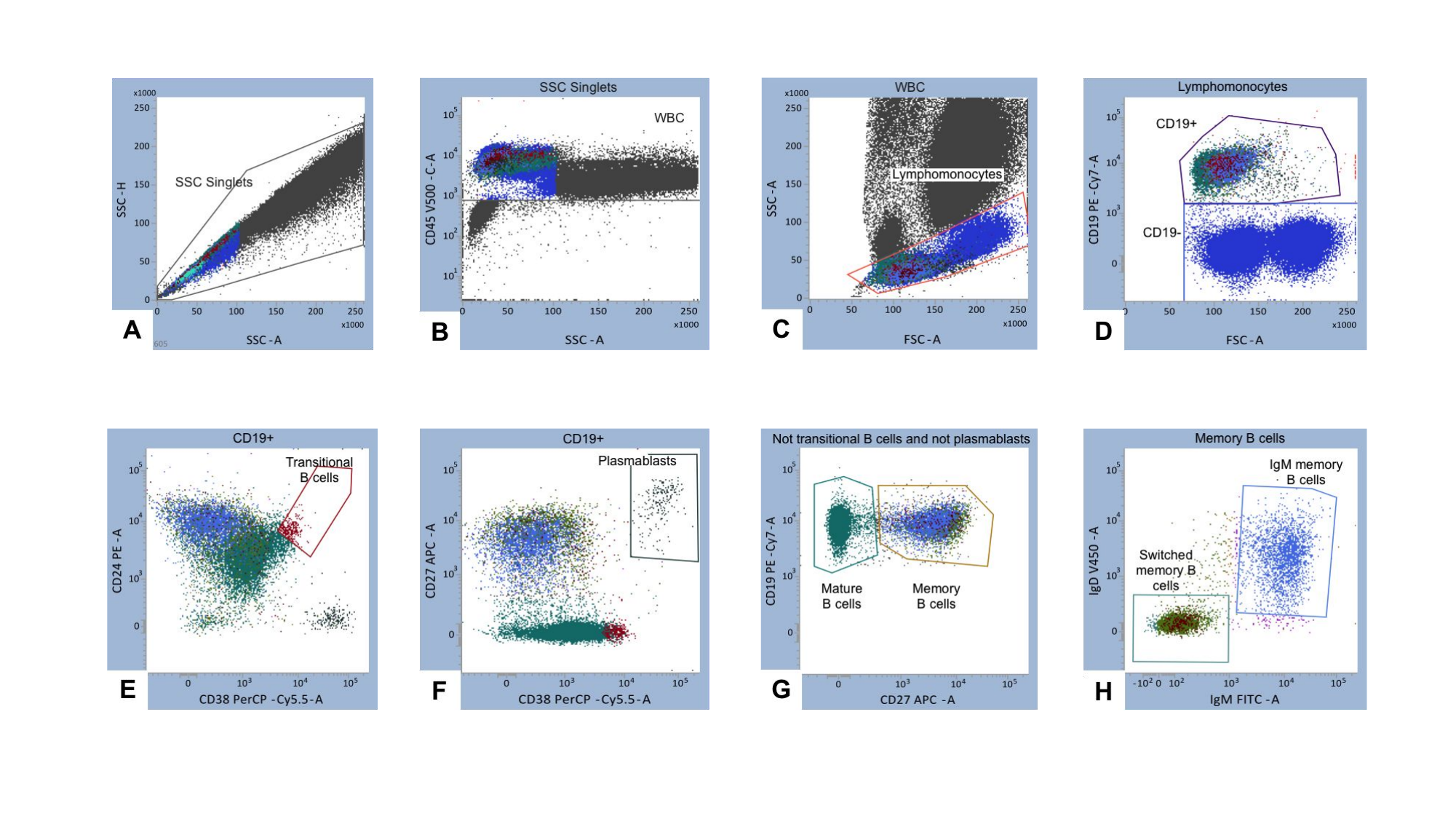

Supplement: Supplementary file 2 — Supplementary Figure 1. [file 41598_2020_77945_MOESM2_ESM.pptx]

## Slide 1
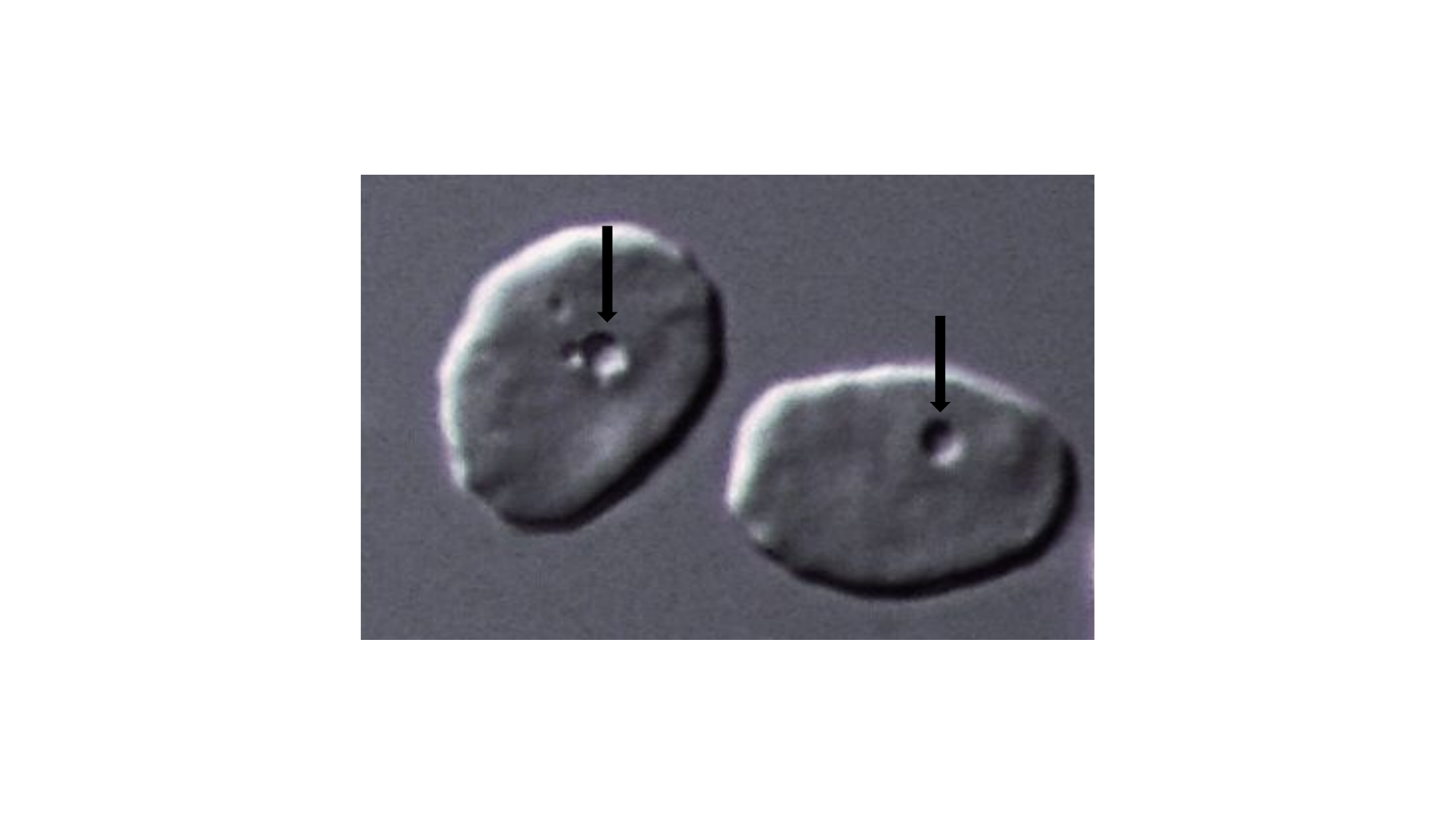

Supplement: Supplementary file 3 — Supplementary Figure 2. [file 41598_2020_77945_MOESM3_ESM.pptx]
